# Supplementary material for: The effects of language and emotionality of stimuli on vocabulary learning
Source: PLoS One. 2020 Oct 7;15(10):e0240252. doi: 10.1371/journal.pone.0240252 (PMC7540870; doi:10.1371/journal.pone.0240252)
Supplement: S1 Fig — Each description was given followed by the questions “How positive or negative is the description of this object?” [¿Cómo de positiva o negativa es la descripción de este objeto?] and “How intense is the description of this object?” [¿Cómo de intensa es la descripción de este objeto?] with a clarification underneath saying “Please select how intense the emotional activation you feel is, regardless of whether the description is positive or negative” [Por favor selecciona cómo de intensa es la activación emocional que sientes, más allá de que la descripción sea positiva o negativa]. After each question there was a dropdown menu. In the valence question the answer options were very negative, somewhat negative, neutral, somewhat positive, and very positive [muy negativa, algo negativa, neutral, algo positiva, muy positiva]. For the arousal question, the options were: not at all intense, a little intense, somewhat intense, very intense, extremely intense [para nada intensa, un poco intensa, bastante intensa, muy intensa, extremadamente intensa]. (PDF) [file pone.0240252.s001.pdf]

ailo

Esto es un ailo. El ailo es un conjunto de pirámides usadas para varios juegos familiares divertidos. Los juegos ailo requieren cooperación, por lo que son fantásticos para fortalecer la unión familiar, así como para conocer mejor a amigos. El ailo es un juego.

*
¿Cómo de positiva o negativa es la descripción de este objeto?

Seleccione una de las siguientes opciones

Por favor escoja...

*
¿Cómo de intensa es la descripción de este objeto?

Seleccione una de las siguientes opciones

Por favor escoja...

Por favor selecciona cómo de intensa es la activación emocional que sientes, más allá de que la descripción sea positiva o negativa.
