# Supplementary material for: The effects of language and emotionality of stimuli on vocabulary learning
Source: PLoS One. 2020 Oct 7;15(10):e0240252. doi: 10.1371/journal.pone.0240252 (PMC7540870; doi:10.1371/journal.pone.0240252)
Supplement: S1 Table — The following is a list of all the pseudowords used for the main tasks—learning and testing—by type, i.e. whether they were used as learn/test stimuli or as foils. Reported are the average token-type bigram frequencies for the word as reported by B-Pal [40] and N-Watch software [41]. (PDF) [file pone.0240252.s002.pdf]

S1 Table

Pseudo word	Spanish Bigram Frequency	English Bigram Frequency	Type
ailo	802.14	1027.36	Test
bairel	136.64	458.07	Test
bangel	417.21	747.24	Test
chasus	64.96	554.18	Test
chefio	244.79	350.07	Test
chivel	115.46	667	Test
condrel	462.11	940.72	Test
congril	601.85	688.47	Test
dela	801.25	1359.98	Test
drique	1117.86	333.79	Test
faisa	285.36	339.67	Test
fauril	239.32	587.51	Test
flevel	215.39	351.09	Test
fortor	632.14	675.91	Test
geda	1076.43	56.78	Test
gimus	58.3	302.36	Test
hertor	433.82	525.58	Test
joba	35.36	173.57	Test
lairul	35.11	331.5	Test
leina	303.04	1854.14	Test
lusel	582.63	341.12	Test
meldo	885.04	273.43	Test
moge	123.1	1458.23	Test
mortul	525.32	669.33	Test
mova	137.68	1510.15	Test
nedel	596.38	807.4	Test
pabel	1073.17	506.22	Test
pactur	301.18	416.67	Test
pexta	823.35	90.23	Test
ploca	492.28	654.14	Test
purcal	539.82	725.94	Test
remin	177.19	466.68	Test
rofa	130.48	441.6	Test
sasto	1715.8	321.13	Test
sedin	721.21	591.94	Test
septur	214	526.3	Test
sictur	122.46	415	Test
sogo	837.56	1011.4	Test
surtal	482.46	732.57	Test
temun	480.85	171.99	Test
testor	366.21	570.82	Test
trequi	644.96	266.43	Test
usil	21.19	293.04	Test
vepa	128.27	634.17	Test
vimo	1870.3	707.79	Test
vinis	536.83	401.46	Test
aptor	779.46	221.46	Foil

aslos	775.22	206.21	Foil
babil	482.14	389.33	Foil
bagle	311.47	635.4	Foil
bedil	527.46	660.59	Foil
bepo	136.96	1220.07	Foil
blusia	300.86	157.53	Foil
brolo	286.43	798.3	Foil
cangul	635.68	744.06	Foil
ceren	521.12	938.77	Foil
deto	551.61	1020.02	Foil
echel	247.81	578.26	Foil
ertur	21.07	28.7	Foil
gitus	103.93	249.28	Foil
grotia	445.39	479.93	Foil
honsor	462.14	492.79	Foil
hutin	135.22	549.7	Foil
insoin	94.11	408.18	Foil
jatus	127.77	390.83	Foil
jeno	534.52	523.01	Foil
jomo	3005.6	1261.01	Foil
jusa	570.48	1337.38	Foil
launal	317.21	613.22	Foil
mastur	524.86	716.58	Foil
mersin	383.5	521.58	Foil
mertal	674.14	651.21	Foil
mindor	349.46	547.09	Foil
murche	283.21	352.49	Foil
musil	694.55	369.06	Foil
pelpus	165.11	602.75	Foil
peptor	295.11	661.83	Foil
pormel	874.29	798.64	Foil
pusal	490.63	397.73	Foil
reltor	338.21	840.71	Foil
rescel	268.75	854.49	Foil
ringre	592.39	854.97	Foil
runal	858.3	466.16	Foil
sertal	749.5	886.58	Foil
sesun	905.04	268.79	Foil
sirtor	330.93	498.51	Foil
suptor	254.29	362.04	Foil
tafrel	119.43	349.03	Foil
tauno	449.42	760.05	Foil
teltor	313.43	375.76	Foil
betil	204.11	768.11	Foil
bomple	432.29	1677.45	Foil
