# Supplementary material for: The effects of language and emotionality of stimuli on vocabulary learning
Source: PLoS One. 2020 Oct 7;15(10):e0240252. doi: 10.1371/journal.pone.0240252 (PMC7540870; doi:10.1371/journal.pone.0240252)
Supplement: S1 Appendix — (PDF) [file pone.0240252.s003.pdf]

S1 Appendix: ANOVAs and comparison with LME results

Experiment 1

For the Name Matching Task, we carried out a two-way mixed factor ANOVA of language and emotionality on accuracy (see Table 4 for means, standard deviations, and 95% confidence intervals). There, we found a main effect of emotionality (marginal in the by-item analysis), $F_1(1, 41) = 5.93$, $p = .019$, $\eta_p^2 = .126$, $F_2(1, 45) = 3.67$, $p = .062$, $\eta_p^2 = .075$, such that names in positive contexts were matched better. There was also a main effect of language, $F_1(1, 41) = 4.64$, $p = .037$, $\eta_p^2 =.102$, $F_2(1, 45) = 5.97$, $p = .019$, $\eta_p^2 = .117$, such that names in the native language context were matched better. There was no interaction between language and emotionality, $F_1(1, 41) = .01$, $p = .920$, $\eta_p^2 <.001$, $F_2(1, 45) = .060$, $p = .808$, $\eta_p^2 = .001$ (Fig 3a). For the same task, we also carried out a two-way mixed factor ANOVA of language and emotionality on response time. There, we found a main effect of emotionality (absent in the by-item analysis), $F_1(1, 41) = 6.98$, $p = .012$, $\eta_p^2 =.145$, $F_2(1, 44) = 2.56$, $p = .117$, $\eta_p^2 = .055$, such that names from a positive context were responded to faster, but no main effect of language, $F_1(1, 41) = .02$, $p = .883$, $\eta_p^2 < .001$, $F_2(1, 44) = .18$, $p = .672$, $\eta_p^2 = .004$, and no interaction, $F_1(1, 41) = 1.42$, $p = .240$, $\eta_p^2 =.033$, $F_2(1, 44) = .71$, $p = .404$, $\eta_p^2 = .016$ (Fig 3b). (See the Table S1.1 below for a summary of the statistics).

Table S1.1: By participant statistics for each dependent variable by task and independent variable.

Task		Emotionality			Language			Interaction		
		Statistic [*F*(1, 41)]	*p*	η_p^2	Statistic [*F*(1, 41)]	*p*	η_p^2	Statistic [*F*(1, 41)]	*p*	η_p^2
Name Recall	Number	3.06	.088	.069	**5.56**	**.023**	**.119**	3.06	.088	.069
	LD	.193	.662	.005	**5.26**	**.027**	**.114**	.629	.432	.015
Old/New	D’	.489	.488	.012	.287	.595	.007	.763	.388	.018
	RT	2.52	.120	.058	2.32	.135	.054	.012	.915	<.001
Name Matching	Accuracy	**5.93**	**.019**	**.126**	**4.64**	**.037**	**.102**	.010	.920	<.001
	RT	**6.98**	**.012**	**.145**	.072	.790	.002	.022	.883	<.001

Note: **Bold** values indicate statistically significant effects. LD stands for Levenshtein distance, D’ stands for D prime, and RT stands for response time.

The recall task was analyzed following a two-way mixed factor ANOVA of language and emotionality on number of words recalled and found a main effect of language such that participants recalled more words in their native language context, $F_1(1, 41) = 5.56$, $p = .023$, $\eta_p^2 = .119$, $F_2(1, 45) = 24.65$, $p < .001$, $\eta_p^2 = .354$, but no main effect of emotionality (although marginal in both the by participant and by item analyses), $F_1 (1, 41) = 3.06$, $p = .088$, $\eta_p^2 =.069$, $F_2(1, 45) = 3.34$, $p = .074$, $\eta_p^2 = .069$, and no interaction (present in the by-item analysis), $F_1(1, 41) = 3.06$, $p = .088$, $\eta_p^2 =.069$, $F_2(1, 45) = 5.04$, $p = .030$, $\eta_p^2 = .101$.

We also carried out the same ANOVA again using the normalized LD instead of the average number of recalled items and found a main effect of language such that participants produced strings closer to the correct one when words were learned in a native language context (marginal in the by-item analysis), $F_1(1, 41) = 5.26$, $p = .027$, $\eta_p^2 = .114$, $F_2(1, 45) = 3.39$, $p = .072$, $\eta_p^2 = .070$, but no effect of emotionality, $F_1(1, 41) = .19$, $p = .662$, $\eta_p^2 = .005$, $F_2(1, 45) = .191$, $p = .664$, $\eta_p^2 = .001$, and no interaction, $F_1(1, 41) = .63$, $p = .432$, $\eta_p^2 =.015$, $F_2(1, 45) = .008$, $p = .929$, $\eta_p^2 < .001$. Nevertheless, these results should be interpreted with caution as there is a clear floor effect. (See Table S1.1 for a summary of the statistics and Table S1.2 for the 90% confidence intervals of the effect sizes.)

Table S1.2: 90% confidence intervals of the effect sizes for all tasks in Experiment 1.

Task	Measure	Emotionality			Language			Interaction		
		η_p^2	*Lower limit*	*Upper Limit*	η_p^2	*Lower limit*	*Upper Limit*	η_p^2	*Lower limit*	*Upper Limit*
Name Recall	Number	.069	.000	.213	**.119**	**.009**	**.275**	.069	.000	.213
	LD	.005	.000	.087	**.114**	**.007**	**.269**	.015	.000	.121
Old/ New	D'	.012	.000	.112	.007	.000	.097	.018	.000	.128
	RT	.058	.000	.197	.054	.000	.191	<.001	.000	.025
Name Matching	Accuracy	**.126**	**.011**	**.283**	**.102**	**.003**	**.254**	<.001	.000	.021
	RT	**.145**	**.019**	**.305**	.002	.000	.064	<.001	.000	.038

The old/new word recognition task was analyzed using D'. A two-way mixed factor ANOVA was carried out on the effects of language and emotionality on D'. Here, only F_1 is reported given that, because of the design, D' could not be calculated by item. We found no effect of language, $F_1(1, 41) = .29$, $p = .595$, $\eta_p^2 = .007$, emotionality, $F_1(1, 41) = .49$, $p = .488$, $\eta_p^2 = .012$, nor an interaction, $F_1(1, 41) = .76$, $p = .388$, $\eta_p^2 = .018$. Nevertheless, recognition of the words they had seen before was accurate, 75.7% on average (SD=11.8%). When looking at response time, a two-way mixed factor ANOVA showed no effect of language (present in the by-item analysis), $F_1(1, 41) = 2.32$, $p = .135$, $\eta_p^2 = .054$, $F_2(1, 45) = 21.75$, $p < .001$, $\eta_p^2 = .326$, emotionality (present in the by-item analysis), $F_1(1, 41) = 2.52$, $p = .120$, $\eta_p^2 = .058$, $F_2(1, 45) = 5.27$, $p = .026$, $\eta_p^2 = .105$, nor an interaction, $F_1(1, 41) = .01$, $p = .915$, $\eta_p^2 <.001$, $F_2(1, 45) = .56$, $p = .456$, $\eta_p^2 = .012$. The response time analyses should be interpreted with caution as there were main effects by item, but not by participant. (See Table S1.1 for a summary of the statistics and Table S1.2 for the 90% confidence intervals of the effect sizes).

Experiment 2

For the Name Matching Task, we carried out a two-way mixed factor ANOVA of language and emotionality on accuracy. There was a main effect of emotionality, $F_1(1, 53) = 27.35$, $p < .001$ $\eta_p^2 = .34$, $F_2(1, 38) = 8.87$, $p = .005$, $\eta_p^2 = .159$, such that names in positive contexts were matched better, but no main effect of language, $F_1(1, 53) = .47$, $p = .50$, $\eta_p^2 = .009$, $F_2(1, 38) = 1.47$, $p = .233$, $\eta_p^2 = .037$, and no interaction, $F_1(1, 53) = .83$, $p = .37$, $\eta_p^2 =.015$, $F_2(1, 38) = 1.10$, $p = .300$, $\eta_p^2 = .028$ (see Fig 5a). For the same task, we also carried out a two-way mixed factor ANOVA of language and emotionality on response time. There was a main effect of emotionality, F_1 (1, 53) = 44.50, $p < .001$ $\eta_p^2 =.456$, $F_2(1, 38) = 7.70$, $p = .009$, $\eta_p^2 = .169$, such that names in positive contexts were matched faster, but no main effect of language, F_1 (1, 53) = .10, $p = .757$, $\eta_p^2 =.002$, $F_2(1, 38) = .49$, $p = .489$, $\eta_p^2 = .013$, and no interaction, F_1 (1, 53) = .22, $p = .640$, $\eta_p^2 =.004$, $F_2(1, 38) = .29$, $p = .597$, $\eta_p^2 = .007$. (See Table S1.3 for a summary of the statistics and Table S1.4 for the 90% confidence intervals of the effect sizes.)

The recall task was evaluated as in Experiment 1. We carried out a two-way mixed factor ANOVA of language and emotionality on number of words recalled. There was a main effect of emotionality (absent in the by item analysis), $F_1(1, 53) = 5.41$, $p = .024$, $\eta_p^2 =.093$, $F_2(1, 38) = 1.19$, $p = .282$, $\eta_p^2 = .030$, such that names in positive contexts were recalled better, but no main effect of language, $F_1(1, 53) = .92$, $p = .341$, $\eta_p^2 =.017$, $F_2(1, 38) = 1.39$, $p = .246$, $\eta_p^2 = .035$, and no interaction, $F_1(1, 53) = .06$, $p = .803$, $\eta_p^2 =.001$, $F_2(1, 38) = .043$, $p = .836$, $\eta_p^2 = .001$. Then, we calculated the normalized LD for each response as in Experiment 1. We carried out the ANOVA again using the LD and found the same results: a main effect of emotionality (absent in the by item analysis), $F_1(1, 53) =$

7.07, p = .010, $\eta_p{}^2$ =.118, F_2(1, 38) = 1.67, p = .204, $\eta_p{}^2$ = .042, such that participants produced more similar strings when the names were learned in a positive context, but no main effect of language (marginal by participant and significant in the by item analysis), F_1(1, 53) = 3.94, p = .052, $\eta_p{}^2$ =.069, F_2(1, 38) = 6.75, p = .013, $\eta_p{}^2$ = .151, and no interaction, F_1(1, 53) = .16, p = .690, $\eta_p{}^2$ =.003, F_2(1, 38) = .07, p = .787, $\eta_p{}^2$ = .002. (See Table 8 for means, standard deviations, and 95% confidence intervals. See Table S1.3 for a summary of the statistics and Table S1.4 for the 90% confidence intervals of the effect sizes.)

Table S1.3: Statistics for each dependent variable by task and independent variable.

Task		Emotionality			Language			Interaction		
		Statistic [F(1, 53)]	p	$\eta_p{}^2$	Statistic [F(1, 53)]	p	$\eta_p{}^2$	Statistic [F(1, 53)]	p	$\eta_p{}^2$
Name Recall	Number	**5.41**	**.024**	**.093**	.92	.341	.017	.06	.803	.001
	LD	**7.07**	**.010**	**.118**	3.94	.052	.069	.16	.689	.003
Name Matching	Accuracy	**27.35**	**<.001**	**.340**	.47	.498	.009	.83	.365	.015
	RT	**44.50**	**<.001**	**.456**	.10	.757	.002	.22	.640	.004

Note: **Bold** values indicate statistically significant effects. RT stands for response time and LD stands for Levenshtein distance.

Table S1.4: 90% confidence intervals of the effect sizes for all tasks in Experiment 2.

		Emotionality			Language			Interaction		
Task	Measure	$\eta_p{}^2$	*Lower limit*	*Upper Limit*	$\eta_p{}^2$	*Lower limit*	*Upper Limit*	$\eta_p{}^2$	*Lower limit*	*Upper Limit*
Name Recall	Number	**.093**	**.007**	**.225**	.017	.000	.110	.001	.000	.048
	LD	**.118**	**.016**	**.256**	.069	.000	.195	.003	.000	.065
Name Matching	Accuracy	**.340**	**.171**	**.475**	.009	.000	.089	.015	.000	.106
	RT	**.456**	**.285**	**.574**	.002	.000	.057	.004	.000	.072

Note: **Bold** values indicate statistically significant effects. RT stands for response time and LD stands for Levenshtein distance.

Finally, the average score on the attentional check for English was 73.2% (SD = 11.5%) and for Spanish it was 73.5% (SD = 11.0%). We used both a frequentist and a Bayesian paired samples t-test of language on accuracy to verify that participants read equally well in both language conditions. The test showed moderate evidence that the null model was more likely than the alternative model, t_1(53) = .22, p = .829, Cohen's D = .029, BF_{01} = 6.59, *error %* = .001, t_2(38) = .64, p = .525, Cohen's D = .203, BF_{01} = 5.72, *error %* = 9.91 x 10^{-6}.

Comparison between ANOVA and LME results

The consistencies and differences in results between these two analyses warrant some discussion.

Firstly, let us examine the effects of emotionality. This effect appears consistently throughout in the name matching task: positive emotionality improves accuracy in both experiments regardless of the analysis employed. With respect to response time, this effect is less consistent, as it appears reliably in Experiment 2, but not in Experiment 1 (see the by-item analysis and the LME).

With respect to the Recall task, accuracy shows an improvement with positive emotionality somewhat consistently in the ANOVAs, but not at all in the LMEs. It is possible that either this effect was driven by specific participants or items and removed by the random intercepts of the LMEs, or that, given the low accuracy in this task, the LMEs did not have enough power to show an effect. This is further endorsed by the fact that the effect on Levenshtein distance is only present in the by-participant ANOVA of the second experiment. Finally, the Old/New task showed some possible effects of emotionality in response time, present in the by-item analysis and the LME, but did not show up in the by-participant analysis. Together, these results suggest a consistent effect of emotionality on recognition, particularly in associating the object with its name—i.e., the Name Matching task—and a weaker, or possibly absent, effect on recall.

With respect to the effects of language, there is a consistent detrimental effect of foreign language in the recall task. In all analyses and both experiments there is an effect of language on Levenshtein distance—although at times marginal. This effect is also present in accuracy for Experiment 1, but completely absent for Experiment 2. As suggested in the main text, this might relate to difficulty differences between experiments. There is a possible effect of language on response time in the LME analysis carried out in Experiment 2, being completely absent in Experiment 1 and both ANOVAs of Experiment 2. For the Old/New task there is also a possible effect of language on response time, present in the LME and by-item analysis, but absent in the by-participant analysis. All-in-all, these results suggest an effect of language on recall and matching accuracy that increases with the number of items in the task, as well as a possible, but inconsistent effect on response times.

Finally, there is no interaction between emotionality and foreign language in any task or analysis.
