# Supplementary material for: The effects of language and emotionality of stimuli on vocabulary learning
Source: PLoS One. 2020 Oct 7;15(10):e0240252. doi: 10.1371/journal.pone.0240252 (PMC7540870; doi:10.1371/journal.pone.0240252)
Supplement: Attachment — Submitted filename: Review PONE D-20-07595.pdf [file pone.0240252.s004.pdf]

Review of the PLOS ONE Manuscript D-20-07595 – The effects of language and emotionality of stimuli on vocabulary learning

This study presents two experiments that examine the effect of language (L1 vs. L2) and emotionality (positive vs. neutral) on the acquisition of new concepts by means of definitions. The two experiments differ in the number of target concepts being studied as well as the presentation of the words (in one or two sessions) and the characteristics of the tasks (e.g., number of options in matching task). Employing two-way mixed-factors ANOVAS, the authors concluded a significant effect of language when the set of target words to be learnt in one go is larger, and a stable significant effect of emotionality, with more positive words being learnt.

The study is interesting, and the design is well thought through. The authors have collected a commendable amount of data and performed some sophisticated analyses. However, the manuscript in its current form would require some changes before it can warrant publication in PLOS ONE. The most prominent issue with the manuscript is that the general focus of the study is unclear. The authors state in the abstract and the discussion that the examination of emotionality can inform the learning of foreign languages, in particular regarding vocabulary. However, there is very little discussion about what pedagogical applications or implications the results might have for vocabulary teaching and learning. Similarly, the review of literature is constrained to only studies on emotionality, but it does not include any review of CLIL, word learning, semantics or how the results from emotionality research can be applied in foreign language learning and teaching. The authors should provide a clearer storyline for the study before it is ready for publication.

These and other issues are described below in more detail.

Introduction

The introduction section provides a useful and detailed review on the scarce emotionality research, including the limitations of previous studies that the current paper is addressing. However, this section only reviews literature on this emotionality topic. While I understand that this is the main aim of the study, reviewing only this type of research creates a feeling on the reader that this study explores emotionality for the sake of it, rather than to inform a ‘greater’

aim, such as aid foreign language pedagogy or theory. In this sense, I believe that it would be more appropriate to also discuss some vocabulary learning literature, in particular related to CLIL, how learning new concepts in a second/foreign language is different from attaching new labels for a known concept, and how the studies in emotionality can inform it. Given the authors' claims that emotionality can be a tool for word learning, reviewing literature on these topics seems essential in order to provide a clear focus to the study.

In addition, how does the idea of emotionality connect with semantic theory and research on word connotations? Is emotionality something essentially different from it, or are word connotations and emotionality related? It seems to me that these concepts are very highly interconnected, thus the theory and research on acquisition of word connotations/register can offer valuable insights for the discussion of results.

Moreover, on p. 3 the authors refer to emotionality multiple times, but have not yet provided a precise definition of the concept. It would aid the readability of the study if the explanation of what the authors mean by emotionality appeared early on in the paper. Similarly, on p. 4 the authors write "Emotionality also facilitates learning in the native language, with emotional words having lower ages of acquisition". What are these *emotional words*? Are they universally emotional, or do they depend on each learner's experience? The concept and how the authors conceptualise it is not clear for the reader at this point. An early definition at the beginning of the introduction would be needed for readability and clarity.

Methodology

The study presents a sound methodology and carefully-considered design. The stimuli are well matched across conditions, and the analyses sophisticated. Also, the study includes a second experiment that accounts for some limitations in the methodology of the first experiment and expand on its findings.

There are a few areas that require some further consideration or explanation, though.

Firstly, it seems to me that exploring the effect of emotionality in learning words in a second/foreign language should include not only positive vs. neutral words, but also the effect of negative emotionality. The authors explain on p.6 their reason to only test positive and neutral emotionality. However, given that the students are on average 24 years old, and given that the authors aimed at assessing its effect on naturalistic and realistic learning conditions, I

feel that examining negative emotionality would have been appropriate and more informative, as not all words a learner needs to know in a foreign language are positive or neutral. Although this cannot be changed without collecting new data, the authors might want to include a limitations section at the end of the paper addressing this issue, and stating that the study cannot inform about L2 word learning of negatively-loaded words.

Secondly, and more importantly, there are some differences between the design of Experiment 1 and 2 which are likely to have influenced the results but are not considered in the discussion. The second experiment included not only less than half the words to be learnt in a session, but also a half-long filler task as compared to the first experiment (7’ vs 14’). This should be expected to have an effect on the number of items learnt, as well as their retention. But also, Experiment 2 includes a fewer number of tests that learners had to complete, already with less items and after less distraction time. On top of that, the cued name-matching recognition task gave half the time for learners to select the correct name (from 5000ms in E1 to 2500 in E2) and from 2 options instead of 3, increasing the chances of correct guessing without knowing to 50%. While this might increase the reliability of response times, it significantly decreases the reliability of the accuracy of responses. How could all these changes have influenced the results? More importantly, could this have included too many extra factors in experiment 2 that affected the findings? How can we be sure which factors were determinant in these results? These issues should be included in the discussion of the results, or at least in a limitations section.

On p.21 the authors also state that for each item of the matching task there were 2 trials: one with a neutral distractor and one with a positive emotionality distractor. Does this mean that the same participant saw this same item twice in the session, with only two options to choose from, and thus, one of them (the correct one) being invariant in the two trials? If this is the case, this would have given away the answer for that item in the second occurrence, thus affecting the results of the task. In fact, the results on this task were higher in E2 as compared to E1. This methodological choice needs to be explained further, and its effect taken into account when presenting and discussing the results.

Finally, the authors mention in the description of the study, and then repeat in the discussion, that their main focus was on using naturalistic materials and tests. I understand how their study is different from previous studies and that, compared to them, it can be more naturalistic and realistic. However, I am uneasy about that claim for various reasons. First, the study involves a very controlled, lab learning situation, which means it is neither naturalistic nor representative

of either a language classroom or an L2 immersion situation. Second, the target words and concepts that the participants were asked to learn were pseudowords and invented concepts. While I understand that this control was required for method robustness and reliability, this is again not a natural or realistic situation. Finally, learners were only given 15 seconds to learn the new word with the definition, which is not representative of a real learning condition. For these reasons, it is important that the authors hedge these claims about the naturalistic and realistic focus of their study.

Results and discussion

On p. 13 the authors explain that results were categorically the same with and without including the removed participants, with a few exceptions. However, they do not clarify whether the analysis and results reported are those without the removed participants. I assume this is the case, but should be made clear for the reader after that paragraph, since saying that results were the same with and without can lead the reader to believe the removed participants where then included for robustness.

Moreover, the results of how many words were learnt by each participant in each task are presented in tables in percentages. However, they are rarely discussed in terms of achievement. If the authors' main focus in this study is to somehow inform word learning/teaching in a second language, then discussing the results in terms of how many words were learnt in each task and what this suggests regarding the effect of emotionality would be required.

This has been done briefly for the recall task in Experiment 1, where the authors report that less than 2 words out of 46 were answered correctly (p.26). This finding raises another important question. Given that the results of the test showed almost negligible learning even immediately after treatment, can we consider that emotionality is an effective tool to improve vocabulary learning in a foreign-language educational settings, as stated in the abstract? How does this compare to other strategies to facilitate word learning in CLIL contexts? If the main focus of the study is intended to be pedagogical, these are issues that need to be addressed. Given word limits, I would recommend moving some of the analyses to the appendices, and dedicate more space in the study to review relevant literature and discuss these findings in light of the main focus of the study, whether pedagogical or not.

Finally, on p. 27, the authors concluded that "difficulty differences within the task are what drove the language effect is supported by the fact that that participants learned the same materials and carried out the same task in both experiments.". As I commented in the methodology section, this matching task involved many significant changes from E1 to E2 which would have influenced the results. In particular, accuracy would have been better (and indeed was) in E2 because the matching involved only two options, thus 50% chances of correct guessing without knowing the word, as well as the fact that the same item was trialled twice, one with a positive one with a neutral distractor plus the correct word. Thus, this conclusion needs to be hedged.

Other considerations and typos.

Abstract: Programs taught in a foreign language require… → It is unclear what kind of programmes the authors are referring to here. Specify the type of programme, i.e., University programme.

Abstract: in a language they are not proficient in. → in a language they are not *completely* proficient in. Students studying a course in a foreign language should be proficient enough, so I think it is important to be specific here.

Abstract: For clarity, I think it would be appropriate to include in the abstract the number of participants who took part in each experiment, as well as their proficiency level.

Throughout the paper: I would avoid employing the terms native language vs. non-native language and rather use the preferred L1 (mother tongue) L2 (second/foreign language) throughout. It seems particularly important to me in a study that explores the effects of word emotionality!

P. 3: "In cases like Content and Language Integrated Learning (CLIL), new information is also conveyed in a foreign language, adding to the complexity of learning and remembering information that is abstracted from sensory experience. Would limiting experience in that way make emotionality useful even for acquiring new concrete nouns? Kousta et al.'s [1] theory would suggest that emotionality, given its independence from concreteness, would indeed play a facilitatory role in that case." → the ideas in this section are not very clear for the reader. I would recommend that you specify the referent in the second and third sentences, i.e., what

does “*in that way”* mean in sentence 2? CLIL, or abstract sensory experience? The same for *“in that case*” in sentence 3.

P. 4: “i.e., new concepts with their associated *name*, not just learning new labels for known words or remembering known words”. → new concepts with their associated *form*.

P. 5: “The foreign language literature has shown inconsistent effects of emotionality andlittle is known about expanding vocabulary in that language” → Do the authors mean … about expanding vocabulary by means of emotionality in a foreign language? The use of “that language” seems inappropriate here, as there is no reference to any particular language. Also, there is plenty of literature on expanding vocabulary in a second language, just no by means of emotionality. This sentence needs to be more precise.

P. 6: “Participants only did one language condition (Spanish: 23 participants; English: 20 participants)” → L1 condition (i.e., Spanish: 23) and L2 condition (i.e., English: 20 participants).

P. 11: “The learning phase consisted of seeing each object along with its description, one by one.” → *the picture and name of* each object along with its description

P. 11 → In the procedure for experiment 1, it would be important for clarity to specify how long the learners took in completing the learning phase. Similarly, in the procedure for experiment 2, it should be specified whether the learning task also allowed 15 seconds for participants to learn each word.

P. 14 → Given that the study aims to show how emotionality can help word learning in a second language, the mean raw score for the results of each task should be reported for clarity, in order for the reader to be able to evaluate the effectiveness of the tasks in real terms.

P. 20: “Participants were not significantly different (*p*’s > .05) from those in Experiment 1 on any of the variables: age, gender, education level, student status, verbal and nonverbal IQ (Kbit—Kaufman & Kaufman, 2013), age of acquisition of English, and Spanish and English LexTALE [22,23].” → This claim would be better supported by including a table showing the lack of sig different between the samples across the various factors.

P. 27, line 552: "…language effect is supported by the fact ***that that*** participants learned…" remove one *that*.

P. 28: "more stable effect that can be taken advantage of regardless of other variables—such as task demands, difficulty, language (as we discuss below), or amount of information." → this has been shown only for some tasks, two languages and two sets of target words with two different levels of information. Thus, the statement should be hedged, specifying that it seems to be a stable effect for the tasks, languages and amounts of info as they were tested/designed in this study.

P. 29: "in the first encounter with a word or concept, the emotional context around it has the same effect in the foreign as in the native language" → I feel that this statement needs to be hedged. This has only been shown in a descriptive context situation and in written format, while first encounters with a concept in the L1 situation are typically embedded in greater context. We do not know if this would be the case in a real situation, but it seems unlikely based on L1/L2 acquisition theory. Thus, for the sake of accuracy, the authors should hedged this general statement, being more specific about the kind of encounter for which this has been shown in the study.

P. 37: "¿Cómo de positive o negative es la descripción de este objeto? → *positivo* o *negativo;* also *intense* → *intensa* (lines 720 and 723).
