# Supplementary material for: The effects of language and emotionality of stimuli on vocabulary learning
Source: PLoS One. 2020 Oct 7;15(10):e0240252. doi: 10.1371/journal.pone.0240252 (PMC7540870; doi:10.1371/journal.pone.0240252)
Supplement: Attachment — Submitted filename: Response to Reviewers.docx [file pone.0240252.s005.docx]

First and foremost, we would like to thank the editor and the reviewers for taking the time to assess our paper and for the very insightful comments and suggestions they have provided. The most significant modifications made to the manuscript relate to further clarifying our aims and definitions, justifying our methodological choices, adding the by item analyses (and the LMEs as a supplementary material), clarifying some limitations of our study, and expanding on its interpretation. The literature covered in the introduction has been expanded and clarifications have been added throughout. In the following pages, we have addressed each of the reviewers’ concerns, one-by-one.

# Reviewer 1

**As well, I find the post-tasks to be shallow indicators of vocabulary learning, and this is not discussed in the current manuscript.**

We thank the reviewer for pointing this out. This has now been clarified both in the discussion and the conclusions. (See lines 621 – 622 and 702 – 706).

**The third post-task, the characteristic task, yields a null result but is barely acknowledged or discussed.**

We thank the reviewer for this comment and appreciate that this might require further clarification. The reason why that task was not reported in more detail is that it was included only as an attentional check and was not perfectly balanced between conditions. The task has been renamed “attentional check” to reflect this. What we mean by that is that the information tested in that task was not equally salient in both emotionality conditions and was not of the same difficulty level between objects. Although the task was not meant to be an assessment of the participants’ memory for the items, if the reviewers would like to see the results as part of the manuscript, we would be more than happy to include them.

These are the results for the attentional check (characteristics task):

For accuracy in Experiment 1, there was a reverse emotionality effect *F_1_*(1, 41) = 14.47, *p* < .001, *η_p_²* = .261, *F_2_*(1, 45) = 11.03, *p* = .002, *η_p_²* = .197, such that participants performed better on the neutral than the positive items, no effect of language (present in the by item analysis), *F_1_*(1, 41) = .16, *p* = .687, *η_p_²* = .004, *F_2_*(1, 45) = 9.97, *p* = .003, *η_p_²* = .181, and no interaction, *F_1_*(1, 41) = 2.55, *p* = .118, *η_p_²* = .059, *F_2_*(1, 45) = .10, *p* = .758, *η_p_²* = .002. For response time, there was no emotionality effect *F_1_*(1, 41) = .009, *p* = .926, *η_p_²* < .001, *F_2_*(1, 45) = 1.15, *p* = .289, *η_p_²* = .026, no effect of language *F_1_*(1, 41) = .009, *p* = .926, *η_p_²* < .001, *F_2_*(1, 45) = .225, *p* = .638, *η_p_²* = .005, and no interaction, *F_1_*(1, 41) = .18, *p* = .674, *η_p_²* = .004, *F_2_*(1, 45) = .025, *p* = .876, *η_p_²* < .001.

For accuracy in Experiment 2, there was an emotionality effect (absent in the by item analysis) *F_1_*(1, 53) = 4.69, *p* = .035, *η_p_²* = .081, *F_2_*(1, 38) = .412, *p* = .525, *η_p_²* = .011, such that participants were more accurate on the positive than the neutral items, no effect of language, *F_1_*(1, 53) = .047, *p* = .829, *η_p_²* < .001, *F_2_*(1, 38) = .050, *p* = .825, *η_p_²* = .001, and no interaction, *F_1_*(1, 53) = .303, *p* = .584, *η_p_²* = .006, *F_2_*(1, 38) = .227, *p* = .636, *η_p_²* = .006. For response time, there was an emotionality effect (absent in the by item analysis) *F_1_*(1, 53) = 30.46, *p* < .001, *η_p_²* = .365, *F_2_*(1, 38) = 2.35, *p* = .133, *η_p_²* = .058, such that participants were faster on positive trials, no effect of language *F_1_*(1, 53) = .290, *p* = .563, *η_p_²* = .005, *F_2_*(1, 38) = .348, *p* = .559, *η_p_²* = .009, and no interaction, *F_1_*(1, 53) = .461, *p* = .500, *η_p_²* = .009, *F_2_*(1, 45) = .210, *p* = .650, *η_p_²* = .005.

**Statistical Concerns:**

**The main concern is that the analysis is done on responses averaged to the participant level. No analysis is done at the item level. This falls short of the statistical state of the art in psycholinguistics, which is to perform a mixed effects analyses of raw trial-level responses, with the inclusion of crossed random intercepts of, at least, subjects and items (Baayen, Davidson & Bates, 2008). Neglecting the issue of variation at the level of items commits the "language as fixed effects fallacy" (Clark, 1973) i.e. the analyst is implicitly assuming that the linguistic stimuli in the experiment exhaust their population, which is trivially false in this case (for a broader discussion in psychology see Yarkoni, 2019). Subject-level averaging of responses comes at the expense of statistical power - which is a concern in paper case, given that the authors test for numerous interactions in a very small sample. As well, the stimuli in this experiment are particularly complex: there are seemingly three sources of potential stimulus level random variance: the images, the pseudowords, and the passages. Therefore, an ideal statistical treatment would have started with random intercepts for all these factors (provided the passages, pseudowords, and images were randomly intermixed). It is worth noting that that while this may seem a significant increase in model complexity, it is not --- amounting to at most 3 additional expended degrees of freedom, while moving to unaveraged data will provide many more degrees of freedom back. Likewise, an analysis on participant level averaged responses does not consider whether the effect of context may vary between subjects. A mixed-effects analysis incorporating a random slope of condition would allow for explicit modelling of this possibility, and achieves a better type I error rate (Barr et al, 2013; Matuschek et al, 2017). Similarly, it is worth noting that a properly specified mixed effect model is also more conservative than subject level averages, despite its additional complexity. Lacking a full mixed-model analysis, I feel skeptical the results would generalize to other passages, objects, or pseudowords, whether the small observed effects would remain statistically significant, and whether the interactions would remain null. Checking the OSF repository given by the authors, it appears no trial level data is present, precluding others from doing a state of the art analysis of their own.**

We thank the reviewer for this suggestion. The F2 analyses have now been added to the manuscript, a supplemental material containing the LMEs is now provided, and the by trial data is now in OSF.

**As well, I'm curious as to why language was manipulated as between subjects in first experiment. This seems to pass up the opportunity to use repeated measurements to allow participants to be their own controls. Considering the authors went to much work to match the participants between groups, as well as gather individual differences measures, choosing not to do a mixed model strikes me as quite a missed opportunity, since it would allow language to be a within-subjects factor, do away with the need for matching participants between groups, and allow for any relation between individual difference measures and condition to be tested. While they do this manipulation in study 2, the choice for experiment one is never justified.**

We thank the reviewer for bringing up this question. For the first experiment, we were concerned that if we mixed the stimuli between languages, we would have spillover effects between languages, which is also why we kept Experiment 2 as a two-session experiment. On the other hand, we were concerned that, after running all of the tasks once, participants would then focus only on the names of the objects—knowing which tasks to expect—and not on the descriptions themselves. After running Experiment 1 between subjects, we got feedback from participants (informally) that the tasks were a complete surprise, so that they were not focusing at all on the lexical items. In addition, we felt like the within-subject verification was necessary and worth the risk of having participants focus too much on the pseudowords. Finally, in Experiment 1, we also wanted to maximize the number of items in order to increase power, but we were limited by the number of easily differentiable objects and pseudowords we could create. By experiment 2, this was no longer an issue as we decided to reduce the number of items per session.

This is not presented as such in the current version of the manuscript, but would be more than happy to include it if the reviewers deem it beneficial.

**What was the distribution of accuracy? Was it normally distributed? Did the authors do any transformations on accuracy in the post tasks?**

We thank the reviewer for this question. Given that the distributions for accuracy were normal for all tasks except Recall, we did not report these tests. No transformations were able to normalize the recall data, but the only difference in results between the parametric and non-parametric test was that for Experiment 2 the effect of emotionality was no longer significant.

**How big are the observed effect sizes and how much should we care about them? Are they practically significant in a way a language educator would care about?**

We thank the reviewer for this question. A paragraph has been added to the discussion section addressing this question in detail. (See lines 663 – 676).

**Conceptual Concerns**

**As is stands, the post tasks administered to the participants are very shallow indicators of word learning. Word learning involves a complex, ongoing acquisition of links between orthographic and phonological forms and meaning, and meaning is acquired to different depths (Perfetti, 2002). Both cued recall and old/new recognition are shallow, more indicative of learning of forms than semantics. A useful post-task would have been a meaning generation task: i.e., to have participants attempt to give the meaning assigned to the object in the passage, given only its name or its picture. The shallowness of these tasks to me also hurts the scientific merit of the conclusions, since knowing if positive context learning of form and semantics would be more valuable. Likewise, it would be interesting to know how positive context may influence production versus comprehension, the current tasks comment only on comprehension.**

We thank the reviewer for bringing up this concern. We do not make claims about acquisition of semantics and talk about acquisition and memory for lexical items. Most studies on foreign language word learning simply ask for direct translations rather than definitions or more complex meaning generation tasks (e.g., Valente, Ferré, Soares, Rato, & Comesaña, 2018; Ferré et al., 2015; Altarriba & Brasnight-Brown, 2011; Ferré, Ventura, Comesaña, & Fraga, 2015). Although It would be interesting to see how deeper, semantics-related processes are affected by emotionality, this is not the goal of the current experiment. Our concern was to observe lexical item integration, in part because it is a necessary step for deeper processing/integration and, in part, because we wanted to see what the effects would be if we maintained testing conditions between languages perfectly equal. Any meaning generation or production task would require different levels of effort between languages, which we wanted to avoid.

**While the authors use the term emotionality in their paper, their manipulation really has more to do with positive valence, due to the lack of a negative condition. As well, although they note emotion captures attention, it is worth noting that, for negative words, additional attention may come at a cost (negative words capture attention and we devote cognitive resources to potential fight or flight responses). Rather than the vague "emotionality" the authors could clarify whether they mean: high arousal, positive valence, or positive and negative valence.**

We thank the reviewer for pointing this out. We manipulated both valence and arousal and therefore use emotionality as a short-hand. This has now been clarified throughout the current version of the manuscript. (See lines 20 – 21, 35 – 36, 144 – 146, 155 – 156, 614 – 616, and 659 – 661).

**The authors claim their stimuli are naturalistic, but it is worth noting that this experiment is not a naturalistic word learning experiment. It is an explicit word learning study, where participants are instructed to learn the words. This is unlike a natural, incidental word learning environment (such an encountering a novel word during passage reading where word learning is not an explicit goal). Rather than call the experimental or stimuli naturalistic, it would be much more correct to say that it attempts to mimic the context of a classroom. Some sections of the manuscript acknowledge this, but others stray into problematic "naturalistic" claims (e.g. line 120).**

We thank the reviewer for bringing up this issue. Learning in the current study is implicit as participants were told to read for comprehension, not to memorize the words. Therefore, participants do encounter the word in a passage where word learning is not the explicit goal, which, as the reviewer points out, is a natural, incidental word learning environment. As we mentioned before, participants informally reported surprise at the word-testing tasks at the end of the experiment, further suggesting they were reading for comprehension and not to explicitly memorize the lexical item. We also further clarified that we meant “naturalistic classroom-like stimuli”. We have clarified in the methods section that learning was incidental, as we realized we had not made explicit that participants were told to read for comprehension rather than for memorization.

**The fact that the third task, the characteristic matching task, was null, goes unremarked and undiscussed in the general discussion. This for me is the most interesting task, because it is the most semantic. This task should actually tap into a different, slightly deeper but still shallow, aspect of word learning. Yet, the authors say it is unimportant and is only done as an "attention check." I find this reporting and framing of that post-task selective and problematic. At minimum it needs to be discussed more thoroughly, since it implies that positive context is only enhancing shallow, form-based aspects of word learning, and less so semantic ones. As well, this is the post task where I would expect 1st or 2nd language to potentially matter most. This task is not revisited at all in the general discussion and it should be.**

We thank the reviewer for this comment. Although we agree that semantics is an important aspect of word learning, it is not the focus of the current study. We aimed to look at the acquisition of new lexical items and were particularly focused on keeping the tasks equal between languages. The reason why this task was not given much attention is that it was kept as an attentional check to make sure participants were reading properly. As this task was added at the end, after the stimuli were created, we were unable to match items properly between conditions, which makes it difficult to extract conclusions from the comparison. In addition, it is the only task that varied between languages, which we explicitly wanted to avoid. Therefore, in depth analyses of this task would not provide a fair picture of the phenomenon. But as we mentioned before, we are more than happy to add these analyses (as reported above) if the reviewers deem it necessary.

# Reviewer #2

**Some researchers have found that valenced words (positive and negative) are processed faster than neutral words in lexical decision tasks (see Ponari et al., 2015; Vinson et al., 2014). The authors even showcase the effectiveness of positive and negative valenced words from a study by Ferré et al., 2015. As such, why did the authors not also include the stimuli with negative descriptions to test for the effects of negative emotionality? Could negatively valenced words/descriptions also provide a similar processing benefit (as with positively valenced words/descriptions) and possibly facilitate learning by participants?**

We thank the reviewer for this comment. As we explain on lines 157 through 163, the reasoning behind the choice to exclude a negative condition was two-fold. First, negative stimuli tend to narrow down the focus on the negative stimuli themselves, even if they are irrelevant for the task. In our current experiments, this would mean focusing on the negative context (i.e., negative words in the description) and away from the information we wanted them to remember (i.e., the new lexical items). Second, the application for this research was meant to be a classroom, where it is often inappropriate to speak about particularly negative and possibly triggering topics.

**Lines 57-59: Run-on sentence; consider breaking up into two sentences.**

**Lines 82-83: This statement is confusing; consider revising.**

**Line 91: This statement is not clear: “Frances et al. [15] took a similar approach to the current one, where participants…” Is “current one” referring to the present study? If so, it should be clarified.**

**Line 102: What is meant by “…expanding vocabulary in that language.”? What are you referring to here?**

**Line 111: Do you have a citation for the statement, “…which is analogous to how we actually learn emotional words.”? This claim is unexplained.**

We thank the reviewer for the stylistic comments and suggestions. These have been incorporated into the manuscript.

**Lines 204-209: Did the researchers control for specific individual differences variables, such as foreign language anxiety, motivation, working memory, etc.? If not, how might these factors have influenced the results of the study?**

We thank the reviewer for this question. We matched participants for linguistic variables such as language level and age of acquisition among others, as well as IQ and working memory. Given that we see the same results in Experiment 2, in a within subjects design, it is difficult to claim that participant variables had a meaningful effect on the emotionality results. With respect to language, it is possible that participant variables could have influenced the results of the first experiment, but given how well the two groups were matched, it is difficult to say. The second experiment, being fully within-subject, is unlikely to have been affected by individual variables.

**Lines 210-213: What was the average length of exposure during the learning phase of each experiment? Did some participants spend longer amounts of time than others studying the objects and their descriptions? If so, what is the likely effect on learning?**

We thank the reviewer for this question. Average reading times have been added to the manuscript. In experiment 1, reading time did correlate positively with performance on the matching task, but not the others. In experiment 2, it did correlate positively with performance (reduced response times, increased accuracy, and reduced Levenshtein distance). Overall, we wanted participants to take the amount of time they felt they needed to understand the texts. Participants may have remembered better the information from texts they took longer to read. Given that reading was self-paced for comprehension, we believe that these analyses are not particularly informative, but are more than happy to include them in the manuscript if the reviewers find them useful.

**Lines 224/417: Why did the researchers choose to arrange the testing items of the two experiments in these particular orders? Could the presence of the first two tests in Experiment 1, for example, have primed participants for the main task (test 3)? In this case, would task randomization have been better suited?**

We thank the reviewer for bringing up these questions and have now clarified our choice in the manuscript. We believe that counterbalancing the order of tasks would have been detrimental as we consider the effects of the different tasks were asymmetrical (as explained in the manuscript). Therefore, we focused on keeping a design that reduced the effects of the earlier tasks on the later ones. (See lines 286 – 291). With respect to priming for the third task, we are not sure how or why that would be the case and, even if it was the case, this priming should theoretically be equally present for all 4 conditions.

**Line 361: Are the overall results from Experiment 1 tenuous, at best, considering the low sample size for each group (N = 20 and N = 23 for the English and Spanish groups, respectively)? This fact could be presented as a limitation or suggestion for improvement during future research.**

We thank the reviewer for this comment. Given that we replicate the results of Experiment 1 with a larger sample in a within subjects design, we believe that our claim is quite strong and the sample size of experiment 1 is not a limitation in the context of Experiment 2.

**Line 525: What do the authors mean by, “…taught in a non-official language.”?**

**Line 568: What do the authors mean by, “…learning new words and concepts may be more costly in a foreign language…”?**

We thank the reviewer for the stylistic comments and suggestions. These have been incorporated into the manuscript.

**Line 597: A variety of concepts and vocabulary presented in the L2 classroom can only be described using negative descriptions (e.g., illness, death, fear, etc.). Based upon the authors’ conclusions in lines 596-598, how can teachers best present these lexical items in the classroom?**

We thank the reviewer for this question. This study is not meant to give direct recommendations to teachers for every case, but rather try to understand the phenomenon of foreign language learning and how the semantic context surrounding a word affects its learning. Nevertheless, we can speculate that changing the way the information is conveyed and contextualized can help put these same terms in a more positive or perhaps hopeful context, thus taking advantage of the effect we describe here. Nevertheless, it is not our intention to provide direct advice, especially not for every case.

# Reviewer #3

**The most prominent issue with the manuscript is that the general focus of the study is unclear. The authors state in the abstract and the discussion that the examination of emotionality can inform the learning of foreign languages, in particular regarding vocabulary. However, there is very little discussion about what pedagogical applications or implications the results might have for vocabulary teaching and learning.**

We thank the reviewer for this comment and have now clarified that the aim of the study throughout. We have emphasized that the application is theoretical and that, although one could extract practical applications, this is not our main goal.

**Similarly, the review of literature is studies on emotionality, but it does not include any review of CLIL, word learning, semantics or how the results from emotionality research can be applied in foreign language learning and teaching. The authors should provide a clearer storyline for the study before it is ready for publication.**

We thank the reviewer for this suggestion and have now added a brief review on CLIL and adult learning in a foreign language. (See lines 53 – 75).

**The introduction section provides a useful and detailed review on the scarce emotionality research, including the limitations of previous studies that the current paper is addressing. However, this section only reviews literature on this emotionality topic. While I understand that this is the main aim of the study, reviewing only this type of research creates a feeling on the reader that this study explores emotionality for the sake of it, rather than to inform a ‘greater’ aim, such as aid foreign language pedagogy or theory. In this sense, I believe that it would be more appropriate to also discuss some vocabulary learning literature, in particular related to CLIL, how learning new concepts in a second/foreign language is different from attaching new labels for a known concept, and how the studies in emotionality can inform it. Given the authors’ claims that emotionality can be a tool for word learning, reviewing literature on these topics seems essential in order to provide a clear focus to the study.**

We thank the reviewer for pointing out this issue. We have now added a brief review on CLIL and adult learning in a foreign language and have clarified the focus of the study. (See lines 53 – 75).

**In addition, how does the idea of emotionality connect with semantic theory and research on word connotations? Is emotionality something essentially different from it, or are word connotations and emotionality related? It seems to me that these concepts are very highly interconnected, thus the theory and research on acquisition of word connotations/register can offer valuable insights for the discussion of results.**

We thank the reviewer for bringing up these issues, but we are not particularly familiar with research on word connotations. Nevertheless, as we suggest in the introduction, research on emotionality presents this as a way of enriching representations (Kousta et al., 2011). Kousta et al. (2011) suggest that the advantage observed for emotional words stems from a particular type of experiential information, more specifically, as being internally embodied through affective states. This presents the benefits of emotionality in parallel to those of concreteness, which are due to the experience through the senses. In this way, the effects of emotionality would probably not be related to the connotation of the word, but rather the richness of the representation that is improved with emotionality.

This clarification is not stated as such in the manuscript, but we would be more than happy to include it if the reviewers deem it beneficial.

**Moreover, on p. 3 the authors refer to emotionality multiple times, but have not yet provided a precise definition of the concept. It would aid the readability of the study if the explanation of what the authors mean by emotionality appeared early on in the paper. Similarly, on p. 4 the authors write “Emotionality also facilitates learning in the native language, with emotional words having lower ages of acquisition”. What are these emotional words? Are they universally emotional, or do they depend on each learner’s experience? The concept and how the authors conceptualise it is not clear for the reader at this point. An early definition at the beginning of the introduction would be needed for readability and clarity.**

We thank the reviewer for this suggestion and have now clarified this in the first sentence of the introduction. (See lines 35 – 37). The emotional words we refer to throughout are those that are on average assessed as more positively valenced and of higher arousal, as defined in the cited database (ANEW) and clarified in the stimulus section.

**Methodology**

**The study presents a sound methodology and carefully-considered design. The stimuli are well matched across conditions, and the analyses sophisticated. Also, the study includes a second experiment that accounts for some limitations in the methodology of the first experiment and expand on its findings.**

**There are a few areas that require some further consideration or explanation, though.**

**Firstly, it seems to me that exploring the effect of emotionality in learning words in a second/foreign language should include not only positive vs. neutral words, but also the effect of negative emotionality. The authors explain on p.6 their reason to only test positive and neutral emotionality. However, given that the students are on average 24 years old, and given that the authors aimed at assessing its effect on naturalistic and realistic learning conditions, I feel that examining negative emotionality would have been appropriate and more informative, as not all words a learner needs to know in a foreign language are positive or neutral. Although this cannot be changed without collecting new data, the authors might want to include a limitations section at the end of the paper addressing this issue, and stating that the study cannot inform about L2 word learning of negatively-loaded words.**

We thank the reviewer for this comment. This has now been added to the discussion as a limitation. (See lines 661 – 662).

**Secondly, and more importantly, there are some differences between the design of Experiment 1 and 2 which are likely to have influenced the results but are not considered in the discussion. The second experiment included not only less than half the words to be learnt in a session, but also a half-long filler task as compared to the first experiment (7’ vs 14’). This should be expected to have an effect on the number of items learnt, as well as their retention. But also, Experiment 2 includes a fewer number of tests that learners had to complete, already with less items and after less distraction time. On top of that, the cued name-matching recognition task gave half the time for learners to select the correct name (from 5000ms in E1 to 2500 in E2) and from 2 options instead of 3, increasing the chances of correct guessing without knowing to 50%. While this might increase the reliability of response times, it significantly decreases the reliability of the accuracy of responses. How could all these changes have influenced the results? More importantly, could this have included too many extra factors in experiment 2 that affected the findings? How can we be sure which factors were determinant in these results? These issues should be included in the discussion of the results, or at least in a limitations section.**

We thank the reviewer for pointing this out and this has now been addressed in the discussion. (See lines 640 – 651).

**On p.21 the authors also state that for each item of the matching task there were 2 trials: one with a neutral distractor and one with a positive emotionality distractor. Does this mean that the same participant saw this same item twice in the session, with only two options to choose from, and thus, one of them (the correct one) being invariant in the two trials? If this is the case, this would have given away the answer for that item in the second occurrence, thus affecting the results of the task. In fact, the results on this task were higher in E2 as compared to E1. This methodological choice needs to be explained further, and its effect taken into account when presenting and discussing the results.**

We thank the reviewer for this question. Given that we had to reduce the number of to-be-learned items for each language, we tried to increase power by increasing the number of trials. We did check any order effects and we found exactly the same results for the first and second trial of each item. We had originally included the between/within emotionality condition variation in case there was an association within the emotional items. This was based on prior literature that suggests that the effects of emotionality are due to increased similarity between emotional items. Nevertheless, given that we found no main effects or interactions with this variable and that this was not a part of our main question, we decided to collapse across condition. This information is not present in the current version of the manuscript, but we would be more than happy to add it, if the reviewers deem it necessary.

**Finally, the authors mention in the description of the study, and then repeat in the discussion, that their main focus was on using naturalistic materials and tests. I understand how their study is different from previous studies and that, compared to them, it can be more naturalistic and realistic. However, I am uneasy about that claim for various reasons. First, the study involves a very controlled, lab learning situation, which means it is neither naturalistic nor representative of either a language classroom or an L2 immersion situation. Second, the target words and concepts that the participants were asked to learn were pseudowords and invented concepts. While I understand that this control was required for method robustness and reliability, this is again not a natural or realistic situation. Finally, learners were only given 15 seconds to learn the new word with the definition, which is not representative of a real learning condition. For these reasons, it is important that the authors hedge these claims about the naturalistic and realistic focus of their study.**

We thank the reviewer for bringing up this issue. We understand that the definition of what is a “natural” learning situation and what are “natural” stimuli is quite complex. For this reason, we have qualified the term naturalistic with “more” throughout the text, as we believe that by comparison, both the situation and the stimuli are more naturalistic than in prior controlled studies. In addition, a comment was included in the discussion alluding to the need for further research on even more naturalistic situations. (See lines 709 – 710)

With respect to the first point, although we agree that the situation is not representative of a classroom with other students, it is quite realistic in that participants were reading about new information, which is in fact what happens in educational settings, be it reading a definition online or in a textbook. With respect to the second point, the fact that words and objects did not exist does not mean they were not naturalistic, realistic, or similar to real words and objects. The pseudowords followed all of the rules of the two languages and the descriptions were realistic and followed all of the laws of the natural world, as well. For these reasons, we do believe that both the situation and the stimuli were realistic.

Finally, learners were forced to read for comprehension for ***a minimum*** of 15 seconds, but they read at their own pace, with an average reading time of 30 seconds per text.

**Results and discussion**

**On p. 13 the authors explain that results were categorically the same with and without including the removed participants, with a few exceptions. However, they do not clarify whether the analysis and results reported are those without the removed participants. I assume this is the case, but should be made clear for the reader after that paragraph, since saying that results were the same with and without can lead the reader to believe the removed participants where then included for robustness.**

We thank the reviewer for this comment and this has now been clarified to avoid confusion. (See lines 307 and 515).

**Moreover, the results of how many words were learnt by each participant in each task are presented in tables in percentages. However, they are rarely discussed in terms of achievement. If the authors’ main focus in this study is to somehow inform word learning/teaching in a second language, then discussing the results in terms of how many words were learnt in each task and what this suggests regarding the effect of emotionality would be required.**

We thank the reviewer for this comment. The main focus of this study is not to inform teaching directly. Nevertheless, the results in practical terms may be interesting/useful and thus a paragraph has been added to the discussion section addressing this issue. (See lines 663 – 676).

**This has been done briefly for the recall task in Experiment 1, where the authors report that less than 2 words out of 46 were answered correctly (p.26). This finding raises another important question. Given that the results of the test showed almost negligible learning even immediately after treatment, can we consider that emotionality is an effective tool to improve vocabulary learning in a foreign-language educational settings, as stated in the abstract? How does this compare to other strategies to facilitate word learning in CLIL contexts? If the main focus of the study is intended to be pedagogical, these are issues that need to be addressed. Given word limits, I would recommend moving some of the analyses to the appendices, and dedicate more space in the study to review relevant literature and discuss these findings in light of the main focus of the study, whether pedagogical or not.**

We thank the reviewer for this comment. In general there are very few studies that look at form recall because of its difficulty. Of the ones that do, the gains in general depend radically on whether learning is explicit or incidental. For example, one study on explicit learning found 18% gains (Brase & Mani, 2017) whereas one on incidental learning had a 4.1% accuracy on recall (Rosa, Tapia, and Perea, 2017). This reflects the overall difficulty of both recall and incidental learning. Nevertheless, this does not imply that there was no learning. In Experiment 1, participants scored on average 46% (21 words) on the name matching task, which is 13% above chance and in Experiment 2, they scored 77% (31 words), 27% above chance.

With respect to emotionality in particular, we have added a paragraph in the discussion elaborating on the size of the effects we found, but it is difficult to compare with the CLIL literature as these often rely on much longer-term training strategies (e.g., 7 sessions throughout 3 months, De Zarobe & Zenotz, 2015) and often rely on testing of material not directly taught in class (e.g., through a general language exam). Still, these tend to find modest improvement (in the example above, there was a 10% improvement with respect to the control group). In a study on content learning (Roussel, Joulia, Tricot, & Sweller, 2017), participants improved performance only 7% or 1.5% (depending on the experiment) by adding the translation of the foreign language text (which was also almost a 5% improvement over the native language text). This suggests that, although gains from learning manipulations in a foreign language tend to be discrete, our results fit within those found in the literature.

Finally, we would like to emphasize that, although our research could lead to practical applications in teaching, our main goal was theoretical. In particular, it was to understand the role of positive semantic context on foreign language learning. For that reason, we are both cautious and reticent to give practical advice. We have emphasized this both in the introduction and the discussion. (See lines 158 – 160, 698 – 701, and 716 – 722).

**Finally, on p. 27, the authors concluded that “difficulty differences within the task are what drove the language effect is supported by the fact that that participants learned the same materials and carried out the same task in both experiments.”. As I commented in the methodology section, this matching task involved many significant changes from E1 to E2 which would have influenced the results. In particular, accuracy would have been better (and indeed was) in E2 because the matching involved only two options, thus 50% chances of correct guessing without knowing the word, as well as the fact that the same item was trialed twice, one with a positive one with a neutral distractor plus the correct word. Thus, this conclusion needs to be hedged.**

We thank the reviewer for this comment. This statement has now been restated as a tentative explanation with a need for further exploration. (See lines 640 – 651).

**Other considerations and typos.**

**Abstract: Programs taught in a foreign language require… → It is unclear what kind of programmes the authors are referring to here. Specify the type of programme, i.e., University programme.**

We apologize for the lack of clarity. We are referring to all kinds of educational programs, not only University programs. This has now been clarified.

**Abstract: in a language they are not proficient in. → in a language they are not completely proficient in. Students studying a course in a foreign language should be proficient enough, so I think it is important to be specific here.**

We apologize for the lack of clarity. We have now clarified this statement.

**Abstract: For clarity, I think it would be appropriate to include in the abstract the number of participants who took part in each experiment, as well as their proficiency level.**

We thank the reviewer for this suggestion and have now included this in the abstract.

**Throughout the paper: I would avoid employing the terms native language vs. non-native language and rather use the preferred L1 (mother tongue) L2 (second/foreign language) throughout. It seems particularly important to me in a study that explores the effects of word emotionality!**

We thank the reviewer for bringing up this issue and have now clarified our choice of terms in the manuscript. We have selected these terms on purpose, given that the participants in this study live in a bilingual community and often speak other languages, either as L1s or L2s. The reason for this relates to emphasizing the status of the language in the community rather than the order of languages learned. The population that was tested for this study is bilingual, with people in this community having one or the other official language as their L1 or L2. Importantly, in this community, order of acquisition does not necessarily correlate with other language characteristics such as dominance or use. Therefore, the distinction we are describing here is between languages that the participant speaks natively and that are officially recognized in the region compared to languages they are less proficient in and that are not the official language of the region; thus the choice of native versus foreign dichotomy. (See lines 54 – 59).

**P. 3: “In cases like Content and Language Integrated Learning (CLIL), new information is also conveyed in a foreign language, adding to the complexity of learning and remembering information that is abstracted from sensory experience. Would limiting experience in that way make emotionality useful even for acquiring new concrete nouns? Kousta et al.’s [1] theory would suggest that emotionality, given its independence from concreteness, would indeed play a facilitatory role in that case.” → the ideas in this section are not very clear for the reader. I would recommend that you specify the referent in the second and third sentences, i.e., what does “in that way” mean in sentence 2? CLIL, or abstract sensory experience? The same for “in that case” in sentence 3.**

**P. 4: “i.e., new concepts with their associated name, not just learning new labels for known words or remembering known words”. → new concepts with their associated form.**

**P. 5: “The foreign language literature has shown inconsistent effects of emotionality and little is known about expanding vocabulary in that language” → Do the authors mean … about expanding vocabulary by means of emotionality in a foreign language? The use of “that language” seems inappropriate here, as there is no reference to any particular language. Also, there is plenty of literature on expanding vocabulary in a second language, just no by means of emotionality. This sentence needs to be more precise.**

**P. 6: “Participants only did one language condition (Spanish: 23 participants; English: 20 participants)” → L1 condition (i.e., Spanish: 23) and L2 condition (i.e., English: 20 participants).**

**P. 11: “The learning phase consisted of seeing each object along with its description, one by one.” → The picture and name of each object along with its description**

We thank the reviewer for these stylistic comments and suggestions. These have now been clarified and incorporated into the manuscript.

**P. 11 → In the procedure for experiment 1, it would be important for clarity to specify how long the learners took in completing the learning phase. Similarly, in the procedure for experiment 2, it should be specified whether the learning task also allowed 15 seconds for participants to learn each word.**

We thank the reviewer for this comment. Average reading times have now been added to the methods section (lines 257 – 258 and 483). It is stated in Experiment 2 that the learning phase was the same as in Experiment 1, which includes the minimum reading time required.

**P. 14 → Given that the study aims to show how emotionality can help word learning in a second language, the mean raw score for the results of each task should be reported for clarity, in order for the reader to be able to evaluate the effectiveness of the tasks in real terms.**

We thank the reviewer for this comment. The effect sizes in practical terms have now been reported and discussed in the general discussion. (Lines 663 – 676)

**P. 20: “Participants were not significantly different (p’s > .05) from those in Experiment 1 on any of the variables: age, gender, education level, student status, verbal and nonverbal IQ (Kbit—Kaufman & Kaufman, 2013), age of acquisition of English, and Spanish and English LexTALE [22,23].” → This claim would be better supported by including a table showing the lack of sig different between the samples across the various factors.**

We thank the reviewer for this suggestion. The t-tests have now been added to the demographics table. (Table 1)

**P. 27, line 552: “…language effect is supported by the fact that that participants learned…” remove one that.**

We thank the reviewer for this comment and this has been rephrased.

**P. 28: “more stable effect that can be taken advantage of regardless of other variables—such as task demands, difficulty, language (as we discuss below), or amount of information.” → This has been shown only for some tasks, two languages and two sets of target words with two different levels of information. Thus, the statement should be hedged, specifying that it seems to be a stable effect for the tasks, languages and amounts of info as they were tested/designed in this study.**

We thank the reviewer for this comment and this statement has now been qualified.

**P. 29: “in the first encounter with a word or concept, the emotional context around it has the same effect in the foreign as in the native language” → I feel that this statement needs to be hedged. This has only been shown in a descriptive context situation and in written format, while first encounters with a concept in the L1 situation are typically embedded in greater context. We do not know if this would be the case in a real situation, but it seems unlikely based on L1/L2 acquisition theory. Thus, for the sake of accuracy, the authors should hedged this general statement, being more specific about the kind of encounter for which this has been shown in the study.**

We thank the reviewer for this comment and this has now been modified.

**P. 37: “¿Cómo de positive o negative es la descripción de este objeto? → positivo o negativo; also intense → intensa (lines 720 and 723).**

We thank the reviewer for this comment and the typos have now been corrected.
