# Supplementary material for: The effects of language and emotionality of stimuli on vocabulary learning
Source: PLoS One. 2020 Oct 7;15(10):e0240252. doi: 10.1371/journal.pone.0240252 (PMC7540870; doi:10.1371/journal.pone.0240252)
Supplement: Attachment — Submitted filename: Response to Reviewers.docx [file pone.0240252.s006.docx]

First and foremost, we would like to thank the editor and the reviewers once more for taking the time to assess our paper and for the interesting points they raised. The most significant modifications made to the manuscript are including the linear mixed effect models in the main text and adding a comparison between the linear mixed effect models and analysis of variance results in the appendix. In the following pages, we have addressed each of the reviewers’ concerns, one-by-one.

# Reviewer 1

**I reviewed a previous version of this manuscript. I find the manuscript better framed than the previous version. In my previous review, I suggested two things: 1) to change the analysis to a linear-mixed effects one, and 2) to better justify numerous choices, particularly the exclusion of the third "attention check" task. Unfortunately, the fears motivating me to prefer the mixed effects analysis appear to have been realized. By-item ANOVAs yield inconsistent effects, and though the mixed model analysis has been done, it is put in the supplementary materials, even though it gives I think much better results.**

**I think the mixed model analysis should be the main one. It is considerably more concise, and actually much more consistent than the by-participant and by-item ANOVAs. As well, I am very distressed that the qualitative differences in effects between these analysis are not remarked on. The reader is merely directed to the supplementary materials, without noting the results are not quite the same. I have created below a table summarizing the results of this analysis.**

**What is worth noting is that there are differences between tasks and experiments, though the overall qualitative trend when significant are identical, which is not the case in the by-item and by-subject ANOVAs. Looking at the mixed model analysis, I am somewhat encouraged, and think the paper makes a good contribution, but it one we should certainly view the findings with many grains of salt. The effects jump around depending on the dependent variable, experiment, and task. The mixed model analysis is much clearer, however.**

We thank the reviewer for these suggestions. In response to this point, we have now changed the manuscript to present the linear mixed effect models as the main analyses and we have provided a comparison between analyses in the supplementary materials (along with the ANOVA results).

**With regards to the "attention check" task, I find the justifications for not analyzing and including the data very poor. If the stimuli are not perfectly matched, then at the very least language can serve as a statistical control, to see if the positivity advantage remains. If there are large differences in the difficulty of the items, this might be revealed by inspecting the residual variance of a random effect of item, and seeing if the random adjustments for items are imbalanced across conditions.**

We thank the reviewer for this comment. Nevertheless, we maintain that this task was not built to be analyzed in more detail and would require a level of discussion that would detract from the main results. We fear that presenting these results would provide an inaccurate picture of any possible effects, as the task varies between experiments and the items varied widely in imageability, length, and importance in the text. This task was conceived and carried out as a rough measure of attention to detract from only focusing on the vocabulary and not as a way to assess learning. Furthermore, in Experiment 1 this task does not involve the learned vocabulary at all, meaning that it completely misses the central theme of the paper, namely, vocabulary learning. For these reasons, we believe that including this analysis would weaken the paper rather than provide further insight.

# Reviewer #3

**Below are some final, minor suggestions that I would like to make before the paper is published:**

**“One of the priorities in the current study is to use naturalistic classroom-type stimuli.”**

**I appreciate that the authors have added classroom-type in response to a comment in the previous round of review regarding how natural stimuli and tasks are. However, I would recommend to further paraphrase this statement slightly, for example: “… to use more naturalistic classroom-type stimuli than in most previous research”.**

We thank the reviewer for this comment and have now clarified this statement further.

**“We hypothesized that new lexical items would be more difficult to learn in a foreign language—even though the words were exactly the same between languages—…"**

**When I read this sentence in the second revision I had to stop and read it again, and I think future readers might experience the same situation. The confusing part was “.. the words were exactly the same between languages”. Maybe this can be paraphrased to say “even though the target words in the stimuli were exactly the same between conditions”.**

We thank the reviewer for this comment and have now clarified this statement.

**“It is worth nothing that our study focuses on….”**

**I think that you meant “noticing” in this statement at the end of the Discussion section. One of the most unfortunate tricks played by autocorrectors that you want being published in a manuscript!**

We thank the reviewer for this comment and have fixed this typo.
